# Combined Gene Expression and RNAi Screening to Identify Alkylation Damage Survival Pathways from Fly to Human

**DOI:** 10.1371/journal.pone.0153970

**Published:** 2016-04-21

**Authors:** Alfeu Zanotto-Filho, Ravi Dashnamoorthy, Eva Loranc, Luis H. T. de Souza, José C. F. Moreira, Uthra Suresh, Yidong Chen, Alexander J. R. Bishop

**Affiliations:** 1 Greehey Children´s Cancer Research Institute, University of Texas Health Science Center at San Antonio, San Antonio, Texas, United States of America; 2 Molecular Oncology Research Institute, Tufts Medical Center and Tufts University School of Medicine, Boston, Massachusetts, United States of America; 3 Department of Cellular and Structural Biology, University of Texas Health Science Center at San Antonio, San Antonio, Texas, United States of America; 4 Departamento de Bioquímica, Universidade Federal do Rio Grande do Sul, Porto Alegre, RS, Brazil; 5 Department of Epidemiology and Biostatistics, University of Texas Health Science Center at San Antonio, San Antonio, Texas, United States of America; CNR, ITALY

## Abstract

Alkylating agents are a key component of cancer chemotherapy. Several cellular mechanisms are known to be important for its survival, particularly DNA repair and xenobiotic detoxification, yet genomic screens indicate that additional cellular components may be involved. Elucidating these components has value in either identifying key processes that can be modulated to improve chemotherapeutic efficacy or may be altered in some cancers to confer chemoresistance. We therefore set out to reevaluate our prior *Drosophila* RNAi screening data by comparison to gene expression arrays in order to determine if we could identify any novel processes in alkylation damage survival. We noted a consistent conservation of alkylation survival pathways across platforms and species when the analysis was conducted on a pathway/process level rather than at an individual gene level. Better results were obtained when combining gene lists from two datasets (RNAi screen plus microarray) prior to analysis. In addition to previously identified DNA damage responses (p53 signaling and Nucleotide Excision Repair), DNA-mRNA-protein metabolism (transcription/translation) and proteasome machinery, we also noted a highly conserved cross-species requirement for NRF2, glutathione (GSH)-mediated drug detoxification and Endoplasmic Reticulum stress (ER stress)/Unfolded Protein Responses (UPR) in cells exposed to alkylation. The requirement for GSH, NRF2 and UPR in alkylation survival was validated by metabolomics, protein studies and functional cell assays. From this we conclude that RNAi/gene expression fusion is a valid strategy to rapidly identify key processes that may be extendable to other contexts beyond damage survival.

## Introduction

Alkylation is a reactive process donating carbon-hydrogen moieties to various classes of biomolecules. Clinically, the most commonly used alkylating agents are nitrogen mustards and other chemotherapeutics such as cyclophosphamide, ifosfamide and temozolomide. On the other hand, prototypical alkylating agents such as methyl-methanesulfonate (MMS) and ethyl-methanesulfonate are advantageous to *in vitro* studies as they do not require bioactivation [1]. Previously, we used RNA interference (RNAi) screening in *Drosophila* cells to identify genes and pathways necessary to survive MMS [1]. Here, we wanted to ask whether it is possible to extend those studies and identify additional pathways that are conserved from fly to mammals by integrating RNAi screening data with MMS-induced gene expression changes.

Towards this goal we used data from our aforementioned *Drosophila* genome-wide RNAi screen to identify genes required to survive MMS [1] and generated complementary gene expression data in fruitfly cells (*Kc167*), mouse embryonic fibroblasts (MEFs) and human cancer cells (MDA-MB231 cells). This rich dataset provided the opportunity to determine the feasibility of fusing phenome and transcriptome data in response to a single stimulus thereby revealing key biological process on a systems level. Noteworthy, this type of complementary analysis has been a longstanding challenge in the field; different publications have reported no significant overlap between the genes that confer a phenotype (the phenome) when knocked out/down and those that are transcriptionally regulated (the transcriptome) in the same context [2, 3]. With our datasets, no gene enrichment (direct ortholog comparison) was seen either across species for gene expressions or within species between gene expression response and RNAi screen data. However, a consistent conservation of alkylation responses across platforms and species was observed at a pathway/process level, and a stronger enrichment stood out when combining the two datasets (RNAi screen plus microarray) prior to analysis. With this, we were able identify the same pathways we previously reported and validated when we evaluated our RNAi screening results [1] as well as additional responses. Our analysis indicated significant changes in DNA-to-protein metabolism (transcription, translation) and proteasome-mediated degradation, glutathione (GSH) biosynthesis and fatty acid metabolism as well as a highly conserved NRF2 and endoplasmic reticulum stress (ER stress)/unfolded protein responses (UPR). The GSH pathway metabolic changes were confirmed by the use of metabolomics in fly and mouse cells. We then went on to evaluate the conservation of response of these pathways and whether modulating them impacted MMS survival in mammalian cells.

## Materials and Methods

### Cell lines and tissue cultures

The *D*. *melanogaster* embryonic cell line *Kc167* was grown in Schneider medium (Invitrogen, Carlsbad, CA) supplemented with 10% heat-inactivated fetal bovine serum (FBS), penicillin and streptomycin at 22°C in a humidified incubator. Primary mouse embryonic fibroblasts (MEFs) were obtained by harvesting 14.5-day C57BL/6J embryos as previously described [1]. Briefly, fetal liver and head were removed and the remainder of the embryo was mechanically disaggregated in plating medium. A suspension of single-cells was plated in DMEM (plus 10% FBS, 100U/mL penicillin and 100μg/mL streptomycin). MEFs were grown for at least three passages before experiments. MDA-MB231 and A549 cells lines were obtained from ATCC and grown in DMEM and RPMI, respectively, supplemented with 10% FBS plus antibiotics in a 37°C humidified incubator at 5% O_2_ and 95% CO_2_ atmosphere. Cells were kept and treated in the exponential phase of growing at 60–70% confluence. Buthionine sulfoximine (BSO, 2 mM) and N-acetyl-cysteine (NAC, 7.5 mM) were from Sigma-Aldrich (USA) and were pre-incubated for 8 h before MMS (40 μg/mL, IC25-50 range at 72 h for all cell lines) treatments.

### Microarray and RNA sequencing gene expression profiling protocols

For microarrays, 7.5x10^6^ *Kc167 Drosophila* cells or 5x10^6^ wild-type MEFs were seeded in T-75 cell culture flask in complete growth medium one day before treatments. On the following day, the medium was replenished with fresh growth medium or medium consisting of MMS at 40 μg/mL. Cells were harvested and pelleted at different times post treatment (0, 1, 8, 24 and 72 h), washed using ice cold phosphate buffered saline (PBS) and the RNA was extracted using the RNeasy kit protocol (Qiagen). RNA integrity was assessed by denaturing formaldehyde agarose gel electrophoresis or by microanalysis (Agilent Bioanalyzer, Santa Clara, CA). Microarray service was provided by Expression Analysis Inc., Durham, NC. Affymetrix GeneChip Drosophila Genome 2.0 Array was used for the experiment performed using *Kc167* cells, and Affymetrix GeneChip Mouse Genome 430A 2.0 Array was used for experiment performed using MEFs. The microarray service provided by Expression Analysis Inc., included group comparison between experimental control and MMS treatment and time course analysis based on permutation analysis of differential expression (PADE). For statistical comparison, each experiment was performed in quadruplicate.

For MDA-MB231 cells gene expression profiling was performed by RNA sequencing protocol using Illumina HiSeq 2000 system (Illumina, San Diego, CA). RNA from 3x10^6^ cells was harvested 8 and 24 h post 40 μg/mL MMS using the RNeasy protocol (Qiagen), and the purity was determined using Agilent 2100 BioAnalyzer. Total RNA samples (1–2 μg) were used for sequencing library preparation according to Illumina TruSeq Total RNA Sample Preparation Guide (Illumina Cat. #: RS-122-2201). Each library was bar-coded and then pooled for cluster generation and sequencing run with 100bp single-end (SE) sequencing protocol. Short read sequences from RNAseq were first aligned to UCSC hg19 genome build using TopHat2 algorithm and then quantified for gene expression by HTSeq [4] to obtain raw read counts per gene and then converted to RPKM (Read Per Kilobase of gene length per Million reads of the library) according to gene length and total mapped read count per sample. Log2-transfromed RPKM measurement were used as gene expression level, and genes with low-read counts (determined by examining read counts in non-exonic regions) were removed. Additionally, genes with low expression (RPKM < 2) in both MMS and untreated conditions were filtered out. Differentially expressed genes (DEGs) at a p<0.05 (T-test control vs MMS-treated) and fold>2 or <-2 in at least one time point were selected as MMS-altered genes. Repository information: GSE57801; http://www.ncbi.nlm.nih.gov/geo/query/acc.cgi?token=apmtuguyplcnbgh&acc=GSE57801

### RNAi screening dataset

Genes whose knockdown caused sensitivity to MMS in Kc167 cells were obtained from our previously published wide-genome RNAi screening in *D*. *melanogaster Kc167* cells as detailed in [1]. This RNAi screening tested 13,826 open reading frames, of which 996 FBgn (FlyBase gene ID, denotes known genes) affected MMS survival; 537 of which were further examined with 202 validating [1]. Microarray detected 12,363 FBgn, with 95% of these had a matched probe with the RNAi screening platform therefore making fusion of RNAi and microarray gene lists feasible.

### Pathway Enrichment Analysis (PEA)

For determination of pathways associated with MMS-induced gene expression changes in fly Kc167 cells, DEGs from 8, 24 and 72 h treatments were combined into a single gene list prior to PEA. In mammalian cells (MEF and MDA-MB231), the DEG list comprised both 8 and 24 h MMS induced gene expression changes. Differentially expressed genes (DEGs) were analyzed using the Ingenuity Systems (IPA, Ingenuity Pathway Analyzer, Qiagen), and enrichment significance was calculated using the right-tailed Fisher Exact Test. Ingenuity analysis was complemented with the publicly available DAVID Bioinformatics Functional Annotation tools set as: i) Pathways; ii) KEGG and Reactome databases. In DAVID, pathway terms in the output were considered as significantly enriched at a p<0.05 and FDR<10%. Uninformative pathway terms such as disease-related or cell-line specific pathways (for example: “Prostate cancer” and “Melanocyte Development and Pigmentation Signaling”) were excluded. Both fly and human orthologues were used to determine pathway enrichments in DAVID and Ingenuity tools as appropriate for each database. In some figures, Ingenuity canonical pathway charts were used to facilitate visualization of MMS-induced alterations with NRF2 and ER stress pathways.

### Gene/Protein Interaction Networks and Landscape Analysis

Protein interaction network was generated using the STRING database [Parameters: i) input: MMS-induced genes and survival hits identified by PEA with each pathway, and converted to human orthologs; ii) Prediction methods: databases, gene fusion, experiments, textmining and co-occurrence; iii) confidence level 0.400 (medium)]. Fly gene/protein interaction networks were built by associating orthologs belonging to NRF2/oxidative stress and UPR-ER stress pathways selected from literature review (Pubmed, Ingenuity and Qiagen databases) as detailed in S3 Table. In summary: i) NRF2-GSH pathway: components of the transcription factor core signaling, NRF2 transcriptional targets, genes involved in GSH synthesis and GSH-mediated detoxification; ii) ER stress/UPR: chaperones and heat shock factors, ER sensors, and ER-to-Nucleus signaling transducers. Briefly, the network was generated using the STRING database [Parameters: i) input: fly orthologs; ii) Prediction methods: databases, gene fusion, experiments, textmining and co-occurrence; iii) confidence level 0.700 (high)]. Network parameters were saved and the links (interaction strength) were handled by Medusa software [5]. Nodes colors include information on MMS-induced changes in gene expression (microarray) and gene survival role in RNAi screening. Interactomes were further analyzed by ViaComplex software [6], which plots the gene expression (means of each replicates per gene) value over the network/interactome, and distributes the microarray signal through network objects (nodes and links), thus building Z-axis/3D topology landscapes.

### Metabolomics

Five replicates of untreated controls or 40 μg/mL MMS-treated *Kc167* cells were harvested after 8 and 24 h incubation. The cell number was adjusted to 9x10^7^ and provided as a 100 μL of packed cell pellet for GC/MS and LC/MS metabolic profiling by mView service provided by Metabolon. Data were statistically analyzed using Welch's Two Sample t-Tests at a p<0.05 significance.

### Cell viability

Cell viability was assessed using CellTiter-Glo kit (Promega) following manufacturer’s instructions.

### Western blot

Protein lysates were prepared using RIPA buffer containing 1 mM PMSF, 1 mM sodium orthovanadate, 1 mM NaF, and 30 μL/mL aprotinin. The proteins (20–30 μg) were resolved in SDS-PAGE, electro-transferred onto nitrocellulose membranes (Hybond-ECL, GE Healthcare) and blocked with 5% BSA. Primary antibodies (1:1000 dilution) included NRF2 (D1Z9C), BiP/GRP78 (C50B12) and CHOP (L63F7) from Cell Signaling; beta-actin (ab8227) from Abcam and ATF3 (C19) from Santa Cruz. After secondary antibody incubation (1:3000, 2 h), the proteins were detected using Lumiglo substrate (Cell Signaling Technology, CA).

### Small interference RNA (siRNA)

The siRNA duplexes (30 nM final concentration) targeting human NRF2 (sc-37030), KEAP1 (sc-43878) and scrambled siRNA-A controls (sc-37007) were obtained from Santa Cruz Biotechnology. Reverse transfections were performed using the Lipofectamine RNAiMAX Reagent (Invitrogen) following manufacturer’s instructions, and protein knockdown was confirmed by immunoblot 48 h post transfection.

### NRF2 reporter gene assay

Cignal Antioxidant Response Reporter kit (Qiagen, USA) was used to address NRF2 transcriptional activity via Antioxidant Response Elements (ARE) activation. Cells were transfected with a mixture of an ARE-driven firefly luciferase and constitutively expressing Renilla-luciferase constructs (40:1 ratio) for 24 h. Afterwards, the cells were treated with alkylation for 8 h and kept for additional 16 h in drug-free culture medium prior to assessment using the Dual-Luciferase® Reporter Assay (Promega). When used, siRNAs were transfected 48 h before ARE-luciferase assessments.

### Plasmid Construct Overexpression

The pcDNA3.1(+)-GRP78/BiP (plasmid 32701, from Dr. Austin; abbreviated as GRP78-pcDNA3) were obtained from Addgene. Cells were transfected with Lipofectamine 3000 (Invitrogen) and kept for 24–36 h for protein expression (validated by Western blot; data not shown). The pCDNA3 and pCDNA3-EGFP plasmid constructs were used as empty vector and transfection efficiency controls, respectively.

## Results

### Gene expression and RNAi screening data do not display extensive overlap at a gene or pathway level

**Fig 1:**
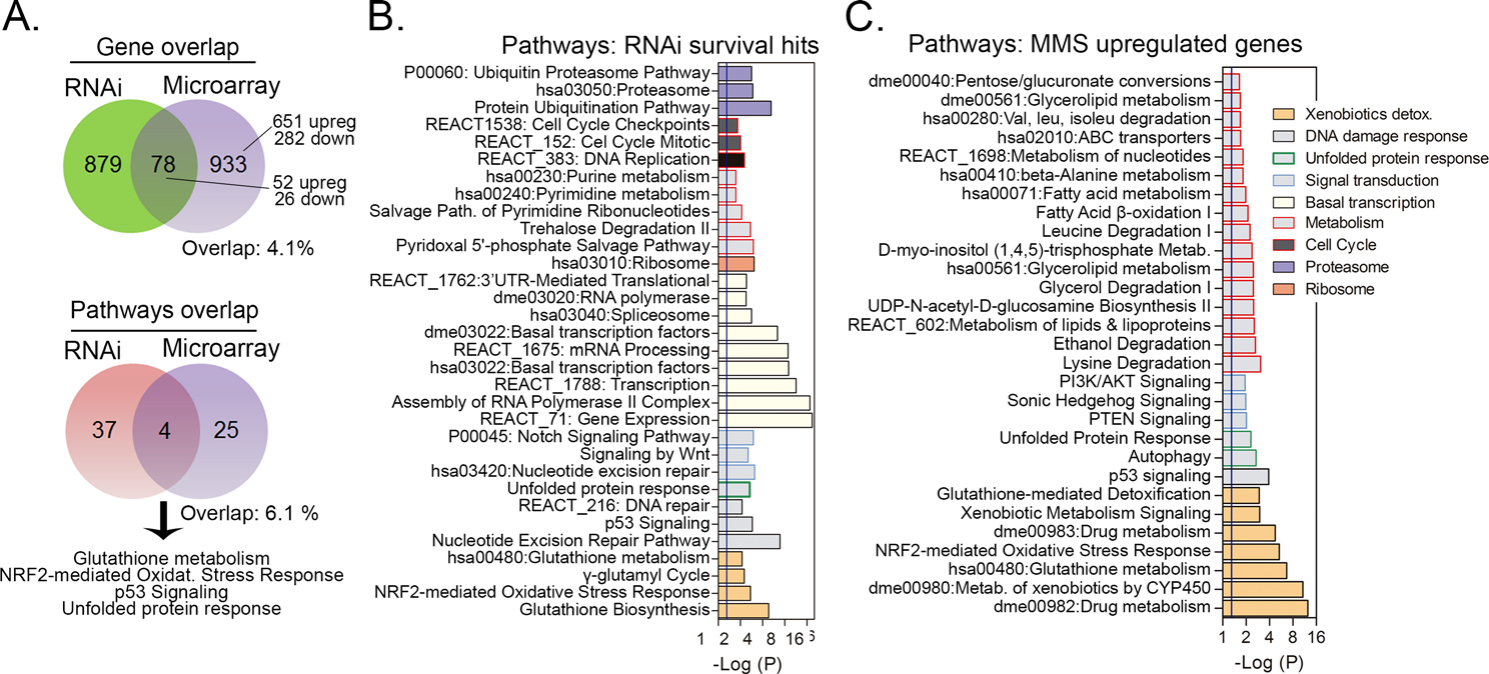
Gene level overlap and PEA showing the cellular processes associated with RNAi hits and gene expression alterations in MMS-treated Kc167 cells. (A) Venn diagrams showing the overlap between survival hits (from RNAi screening) and alkylation altered gene expressions (from microarrays) in MMS treated *Kc167* cells. For gene level overlaps, RNAi hits were obtained from [1] and only those with matched microarray expression changes in at least one time point (8, 24 and 72 h) were used. Microarray gene numbers include both MMS up (703) and downregulated (308) genes as detailed in Results section. Pathway comparisons only show pathways associated with MMS upregulated gene expressions compared to RNAi hits. The 26 RNAi hits with downregulated expressions (S1 Table) were not included in the PEA. (B-C) Antilog P-value representation of the pathways associated with the MMS survival RNAi hits and MMS upregulated genes as predicted by PEA. Pathways are shown as grouped into major biological concepts. Detailed information of differentially expressed genes and PEA are provided in S1 and S2 Tables, respectively.

Our initial goal was to determine whether it is possible to compare gene expression and RNAi screen data to identify genes or pathways for subsequent validation. Previously we had identified 996 FBgn/genes in an RNAi screen for MMS survival using *Drosophila* cells (pre-validation) [1]. 957 of these RNAi hits could be mapped to the genes represented on the Affymetrix GeneChip Drosophila Genome 2.0 Array. MMS exposure resulted in 1011 significant gene expression changes (703 up and 308 down regulated) over 8, 24 and 72 h treatments (Fig 1A), with different gene sets showing transient or prolonged mRNA altered expression (detailed in S1 Fig and S1 Table). For example, two gene subsets, GSH metabolism (*Gclc*, *Gclm*, *GstD2*, *GstD3*, *GstD4*, *GstD5*, *GstD6*, *GstD7*, *GstD10*, *GstE3*, *GstE7*, *GstE9*) and heat-shock proteins (*Hsp23*, *Hsp26*, *Hsp27*, *Hsp67Bc*, *Hsp70Bc*), showed upregulation following short exposure to alkylation (8–24 h) but not at 72 h treatment (S1 Fig).

Despite the comparable number of resultant changes, we observed that most of the MMS survival hits showed no alteration in gene expression (Fig 1A). Only 78 (52 up and 26 down regulated) out of 957 RNAi screen hits displayed altered gene expression in at least one time point in Kc167 cells (Fig 1A, detailed in S1B–S1D Fig and S1 Table). Comparing the genes identified by RNAi or gene expression revealed only a minimal overlap (4.1%, e.g. 78/1890 genes identified by both assays, Fig 1A; Fisher’s exact, P ≤ 0.73). This result indicates that most of the genes that confer survival are not dynamically expressed and concurs with prior reports of no enrichment between these platforms [2, 3]. In the RNAi/microarray overlap, 29 out of 52 RNAi hits with upregulated expression were previously tested and 21 validated (21/29, e.g. 72% validation), including *p53*, the GSH metabolic enzymes *Gclc* and *GstE3*, the chaperones *Hsp70Bc*, *Hsp70Bb*, and the thioredoxin *TrxT* (from [1]). Of note, this is about twice the validation rate observed when just examining RNAi screen hits alone [1]. We also noted that of the 78 overlapping genes, MMS-induced expressions displayed a robust fold-change in microarrays while MMS downregulations were closer to the fold-change cut-off (S1C Fig; detailed in S1 Table). Because of this observation the pathways associated with these downregulated genes were not further investigated. Of note, these 52 up- and 26 downregulated genes do not consistently enrich any particular pathway terms when analyzed by PEA.

Previously we had noted that there was a significant conservation for the pathways necessary to survive MMS across species, if not at an individual gene level [1]. We therefore evaluated whether we might see a similar conservation at a pathway level, which was not present at an individual gene level. Pathway enrichment analysis (PEA) suggested that a majority of the genes altered in each platform participate in different cellular processes, which might explain the lack of overlap observed at a gene level (Fig 1B and 1C, see PEA genes-in-pathway details in S2 Table). Again, no significant overlap (6.1%; 4/66 pathways, Fig 1A) was observed in the pathways identified from either the expression or RNAi screen data (Fig 1A; Fisher’s exact P ≤ 0.44). This occurred because while several RNAi survival hits were associated with some housekeeping processes such as “basal transcription”, “ribosome”, “proteasome”, “cell cycle” as well as DDR responses “Nucleotide Excision Repair (NER) and p53 signaling” and some signal transduction pathways such as “Notch signaling” (most of which we previously validated [1]), the MMS-induced gene expressions involved a different set of genes belonging to a variety of metabolic pathway covering, mainly “GSH metabolism”, “GSH-mediated detoxification”, “Xenobiotic detoxification”, “nucleotides”, “fatty acid” and “amino acid” metabolism (Fig 1B and 1C, detailed in S1 Table). Four pathway terms were enriched by both platforms (Fig 1A); “glutathione metabolism”, “NRF2-mediated Oxidative Stress Response”, “p53 signaling”, “unfolded protein response (UPR)” being the most enriched responses (Fig 1B and 1C; genes-in-pathway are shown in S2 Table), indicating that they are both dynamically expressed in response to MMS and essential for its survival. MMS downregulated genes were associated with 9 pathways terms that are related to DNA replication machinery including DNA polymerases and the MCM (minichromosome maintenance protein complex) family of proteins (this included fly orthologs for *POLE*, *POLA1*, *POLA2*, *MCM2*, *MCM3*, *RNASEH2A*, *MCM4*, *MCM5*, *MCM6*, *RFC5*, *PRIM1*, *DNA2*, *MCM7*, *POLE2*, *POLD2* (S2 Table)).

### Fusion of gene expression and RNAi data improves pathway detection

**Fig 2:**
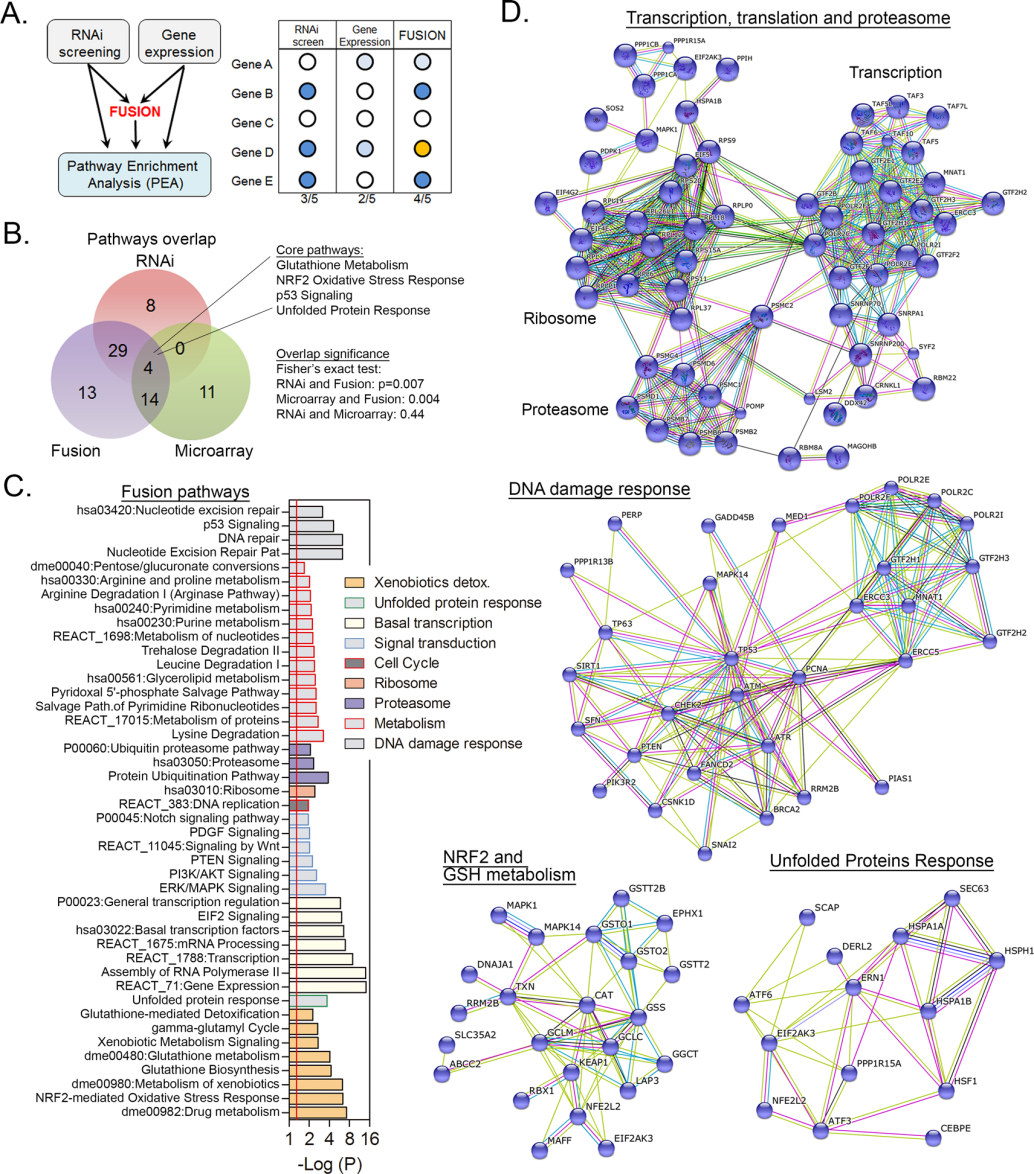
Microarray/RNAi data fusion. (A) Schematic representation of the fusion strategy for MMS-induced gene expression changes and RNAi survival hits followed by Pathway Enrichment Analysis (PEA). (B) Pathway level overlap of MMS-induced survival responses from analysis of microarray, RNAi survival hits and fusion (microarray+RNAi hits) gene lists. (C) Antilog p-value representation of the pathways identified by PEA. Pathways are grouped into major biological processes, and detailed results are described in S2 Table. (D) Protein-protein interaction networks of MMS induced genes and hits with the “transcription, translation and proteasome”, “DNA damage response” and “NRF2” and “UPR” pathways in *Kc167* cells. Networks were developed by inputting into the STRING database both genes induced by MMS to alter expression and those necessary for survival (RNAi hits; converted to human orthologs) as identified by PEA. Color legends of edges in STRING interactomes denote “experiments” (pink), “databases” (light blue), “co-expressions” (black), “textmining” (lime green) and “co-occurrence” (blue) interactions between two nodes.

Having observed that neither comparing gene expression or RNAi survival at either individual gene or pathway level resulted in extensive overlap across platforms, we went on to test if the union of RNAi hits and gene expression changes (DEGs) could change the results of our analysis or lead to identification of novel responses (schematic in Fig 2A). The fusion strategy entails the combined lists of RNAi hits with DEG lists from the microarray experiments (upregulated genes in this case) prior to running PEA. Pathway terms detected by fusion significantly overlapped with the pathways identified independently by either DEG or RNAi hits; e.g. 33/41 (Fisher’s exact test p = 0.007) and 18/29 (Fisher’s exact test p = 0.004) pathways associated with RNAi hits and MMS upregulated genes, respectively (Fig 2B, detailed in S2 Table and S2 Fig). On the other hand, 8 RNAi and 11 microarray-identified pathways were not detected in the fusion output (Fig 2B). These 8 and 11 pathway terms showed a low enrichment p-value in individual microarray/RNAi analysis (S2 Fig) and were likely below the PEA threshold when the fused gene list was evaluated. In addition, the fusion resulted in 13 additional terms (Fig 2B; and detailed in S2 Fig and S2 Table) which were at the threshold of enrichment (p-value~0.05) or redundant to other pathway terms already noted by microarray or RNAi PEA alone. Based on these results we decided to focus on the most consistent/enriched processes.

The greatest benefit of fusing data is the resulting enrichment in genes-in-pathway numbers detected within many pathways previously identified by RNAi and microarray platforms individually (Table 1, Fig 2B and 2C and S2 Table). In contrast, the comparison between RNAi and gene expression data distinguishes transcriptionally dependent and independent survival responses to alkylation. The increased gene numbers by fusion is especially notable for “NRF2 oxidative stress response”, “p53 pathway”, “GSH metabolism” and “UPR” (Fig 2C, Table 1 and S2 Table). This result highlights that these pathways have some genes that are essential for survival and others that may dynamically expressed in response to MMS but that these may not be the same genes. To illustrate this relationship mapping these results onto protein-protein interaction networks from the STRING database reveal that MMS survival genes (hits and differentially expressed) condense into major MMS response modules (Fig 2D; DDR, transcription/ribosome, proteasome, NRF2-GSH and UPR).

**Table 1:** Fusion of RNAi hits and microarray expression changes improves pathway detection. Table showing the number of genes-in-pathway associated with microarray DEG (upregulations), RNAi survival hits or fused gene lists in Kc167 cells treated with MMS. P-value of Fisher-exact test (one-tailed) comparison of the proportion of MMS altered/survival genes in each platform is also shown. “ND” (not-detected) means a given pathway was not detected or was not significantly enriched at a FDR<10%. Legend: “Total genes”: total number of genes in the reference pathway; “Microarray”: MMS-induced gene expressions; “RNAi screen”: MMS survival hits.

	Altered gene numbers per pathway				Fisher Exact p-value	
Pathway terms	Total genes	Micro-array	RNAi screen	Fusion	Fusion vs Microarray	Fusion vs RNAi
**Microarray and RNAi detected**						
NRF2-mediated Oxidative Stress Response	180	13	12	24	0.041	0.026
p53 Signaling	98	8	11	18	0.028	0.113
Dme0048:Glutathione metabolism	50	18	8	22	0.271	0.002
Unfolded protein response	54	4	6	9	0.11	0.29
Glutathione Biosynthesis	3	1	3	3	0.04	1.00
**RNAi screening and Fusion detected only**						
hsa00230:Purine metabolism	153	ND	12	18	p<0.01	0.17
hsa00240:Pyrimidine metabolism	95	ND	9	13	p<0.01	0.24
EIF2 Signaling	185	ND	18	23	p<0.01	0.25
REACT_71:Gene Expression	350	ND	54	60	p<0.01	0.30
Salvage Pathways of Pyrimidine Ribonucleotides	93	ND	7	10	p<0.01	0.31
Protein Ubiquitination Pathway	255	ND	20	23	p<0.01	0.38
hsa03420:Nucleotide excision repair	44	ND	8	10	p<0.01	0.39
hsa03010:Ribosome	87	ND	11	13	p<0.01	0.41
REACT_1788:Transcription	131	ND	27	29	p<0.01	0.50
P00023:General transcription regulation	38	ND	12	12	p<0.01	0.50
REACT_1675:mRNA Processing	32	ND	13	13	p<0.01	0.50
REACT_11045:Signaling by Wnt	61	ND	9	10	p<0.01	0.50
P00045:Notch signaling pathway	50	ND	8	8	p<0.01	0.50
Nucleotide Excision Repair Pathway	35	ND	9	10	p<0.01	0.50
hsa03050:Proteasome	47	ND	8	9	p<0.01	0.50
hsa03022:Basal transcription factors	35	ND	13	13	p<0.01	0.50
Assembly of RNA Polymerase II Complex	50	ND	19	19	p<0.01	0.50
γ-glutamyl Cycle	15	ND	3	4	p<0.01	0.50
REACT_383: DNA replication	97	ND	11	13	p<0.01	0.50
**Microarray and Fusion detected only**						
Xenobiotic Metabolism Signaling	271	12	ND	21	0.07	p<0.01
REACT_1698:Metabolism of nucleotides	77	6	ND	12	0.10	p<0.01
PTEN Signaling	118	6	ND	11	0.15	p<0.01
hsa00561:Glycerolipid metabolism	45	6	ND	9	0.29	p<0.01
dme00980:Metabolism of xenobiotics by CYP450	64	23	ND	26	0.35	p<0.01
dme00982:Drug metabolism	66	25	ND	28	0.36	p<0.01
dme00040:Pentose and glucoronate conversions interconversions	40	10	ND	12	0.40	p<0.01
Lysine Degradation	5	2	ND	3	0.50	p<0.01
PI3K/AKT Signaling	123	6	ND	12	0.11	p<0.01
**Fusion detected only**						
Amyloid Processing	51	ND	ND	7	p<0.01	p<0.01
REACT_13:Metabolism of amino acids	163	ND	ND	18	p<0.01	p<0.01
Hsa00330: Arginine and Proline Metabolism	53	ND	ND	9	p<0.01	p<0.01
ERK/MAPK Signaling	187	ND	ND	18	p<0.01	p<0.01
PDGF Signaling	77	ND	ND	8	p<0.01	p<0.01
REACT_17015:Metabolism of proteins	217	ND	ND	25	p<0.01	p<0.01

### Dynamics of NRF2 and UPR gene expression regulation and essentiality for surviving alkylation

**Fig 3:**
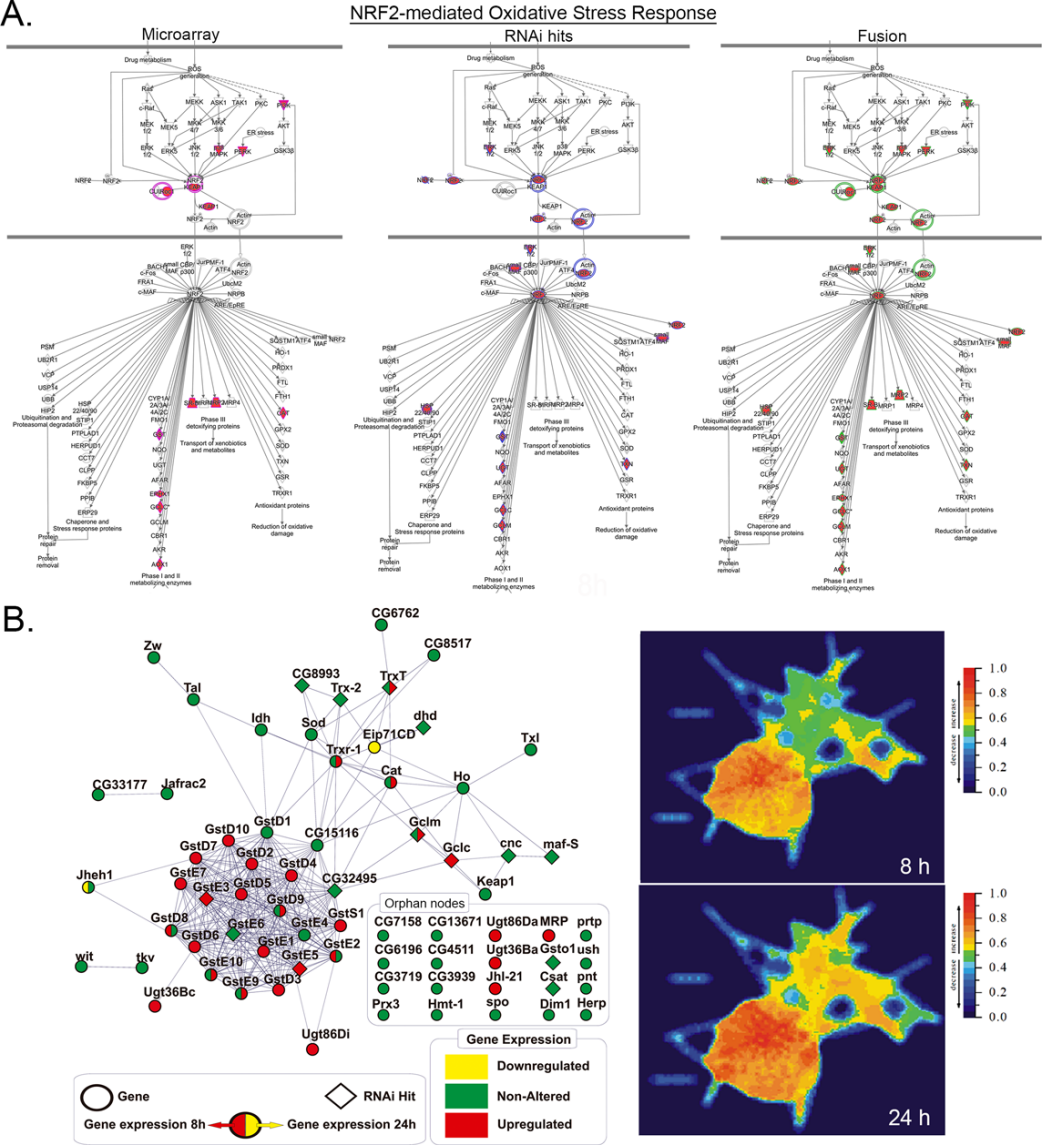
Gene/Protein interaction networks of NRF2-GSH pathway in MMS treated *Drosophila* cells. (A) Ingenuity canonical pathway charts showing gene expression inductions (left), RNAi hits (center) and fusion (right) of MMS responses with the NRF2 pathway. All edges are supported by at least one reference from the literature and stored in the Ingenuity Knowledge Base. Nodes are displayed using various shapes that represent the functional classes of the gene product (square: cytokines; diamond: enzyme; circle into a circle: complex/group; trapezium: transporter; ellipse/oval shape: transcription regulator; triangle: kinase; circle: other) (B) Fusion of gene expression profiles and RNAi screening hits applied to Viacomplex functional networks shows a landscape of overexpressed and lethal components/clusters with the NRF2-GSH pathway in *Kc167* cells treated with MMS for 8 and 24h. The genes used to build NRF2-GSH interactomes, the MMS-induced changes in gene expression and their survival role (from RNAi screen) are described in S3 Table.

**Fig 4:**
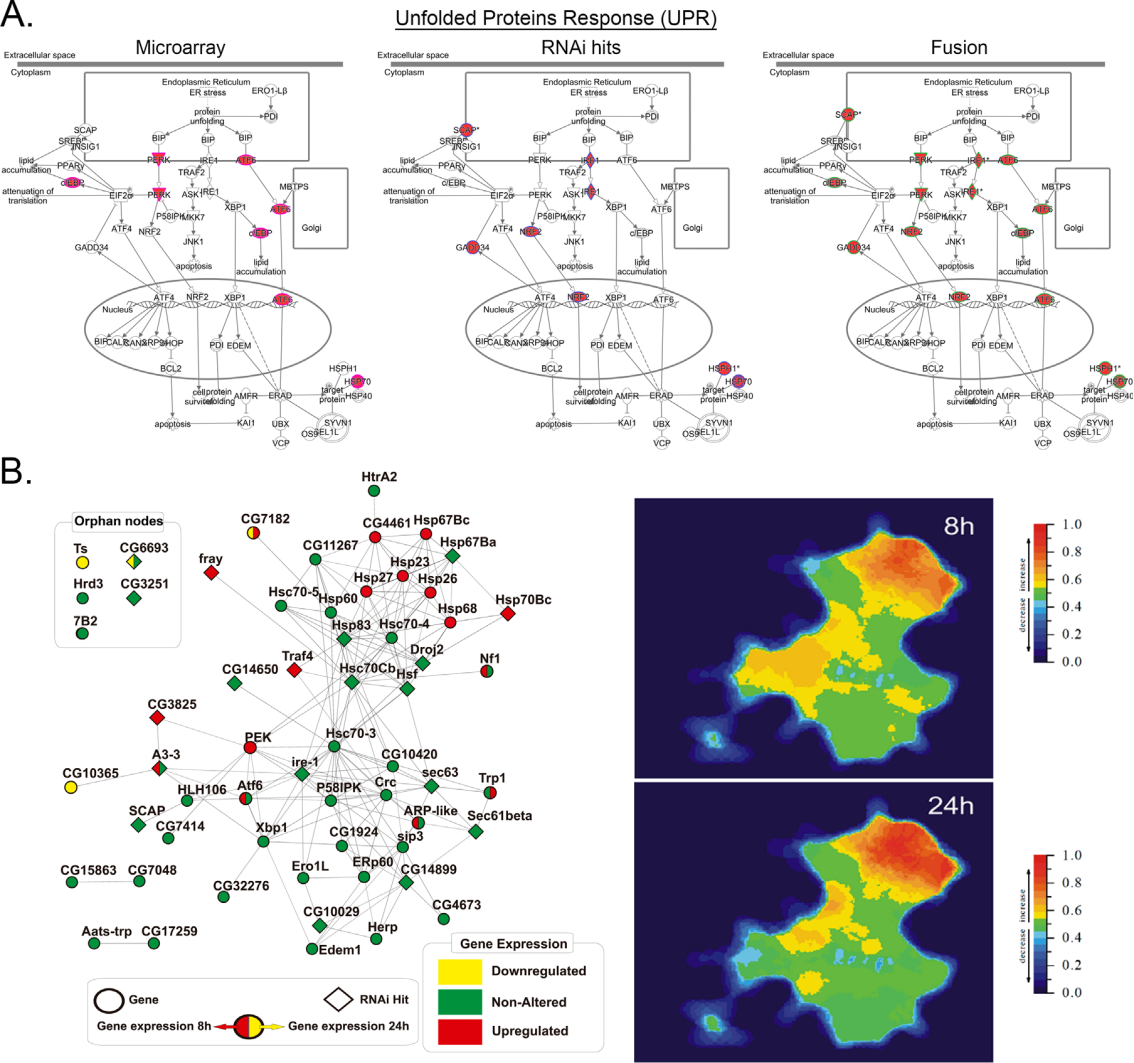
Gene/Protein interaction networks of UPR/ER stress pathway in MMS treated *Drosophila* cells. (A) Ingenuity canonical pathway charts showing gene expression inductions (left), RNAi hits (center) and fusion (right) of MMS responses with the pathway. Details of the edges and nodes are as described for Fig 3. (B) Fusion of gene expression profiles and RNAi screening hits applied to Viacomplex functional networks shows a landscape of overexpressed and lethal components/clusters with the UPR pathway in *Kc167* cells treated with MMS for 8 and 24h. The genes used to build UPR interactome, the MMS-induced changes in gene expression, and their survival role (from RNAi screen) are described in S3 Table.

In our prior *Drosophila* RNAi screening we had validated basal transcription, ribosome, proteasome, NER/p53 DDR and NOTCH as MMS survival responses [1]. Reanalysis of those data and parallel gene expression data with newer bioinformatics tools revealed additional pathways, including NRF2 signaling and UPR. These pathways were further highlighted by additional gene enrichment following the fusion of RNAi hits and MMS-induced gene expressions (Figs 3A and 4A).

Though these processes can be drawn as linear pathways (Figs 3A and 4A), the interactions between components and complexes are more complicated. To determine any particular relationship between the components identified in alkylation response, protein interaction networks were developed for each pathway (Figs 3B and 4B; see S3 Table for NRF2 and UPR pathway gene expressions). Gene expression changes over time within these networks were then examined by ViaComplex analysis. Within the NRF2 pathway a highly connected cluster comprising glutathione S-transferase (GST) orthologs and GSH synthesis enzymes form 8 to 24 h (Fig 3B). Genes such as *GstE5*, *GstE3* (GST family), *Gclc* (GSH synthesis rate-limiting enzyme) and TrxT (thioredoxin), were both essential for survival and up-regulated by MMS. Others, such as the *NRF2* ortholog *cnc* and its transcriptional co-activator *maf-S*, as well as the NRF2 target genes involved in GSH synthesis (*Gclm* and *CG32495/Glutathione synthetase/GSS)* and thioredoxins (*Trx-2*, *CG8993*, *dhd and CG8517*) were not transcriptionally regulated but enhanced MMS toxicity when depleted (Fig 3B and S3 Table).

Similar to our analysis of the NRF2 pathway, we also examine the interaction and dynamic relationship with the UPR pathway. Of note, the *Drosophila* UPR pathway response to MMS appears more dependent upon heat-shock proteins than activation of ER stress mediators as seen in mammalian systems (Fig 4B and S3 Table) [7–10]. MMS up-regulated a well-connected cluster involving *Hsp67Bc*, *Hsp70Bc*, *Hsp*23, *Hsp26* and *Hsp27* chaperones. Knockdown of the key heat shock transcription factor, *hsf* (heat-shock-factor, human HSF1 ortholog), or its direct transcriptional targets *Hsp*83 and *Hsp70Bc*, sensitized cells to alkylation. Hsf regulates a variety of heat shock proteins such as Hsp67Bc [11], a chaperone known to protect against protein misfolding in fly [12, 13]. Of note, HSF1 and HSP12/HSP26 induction were also reported in MMS-treated *S*. *cerevisiae* [14]. Knockdown of some components of classical ER stress machinery also potentiated MMS toxicity in fly cells, including Ire-1 (IRE1alpha ortholog) and the DNAJ chaperones *CG14650* and *CG6693* (Fig 4B and S3 Table).

### NRF2-GSH and UPR are evolutionary conserved alkylation survival programs

**Fig 5:**
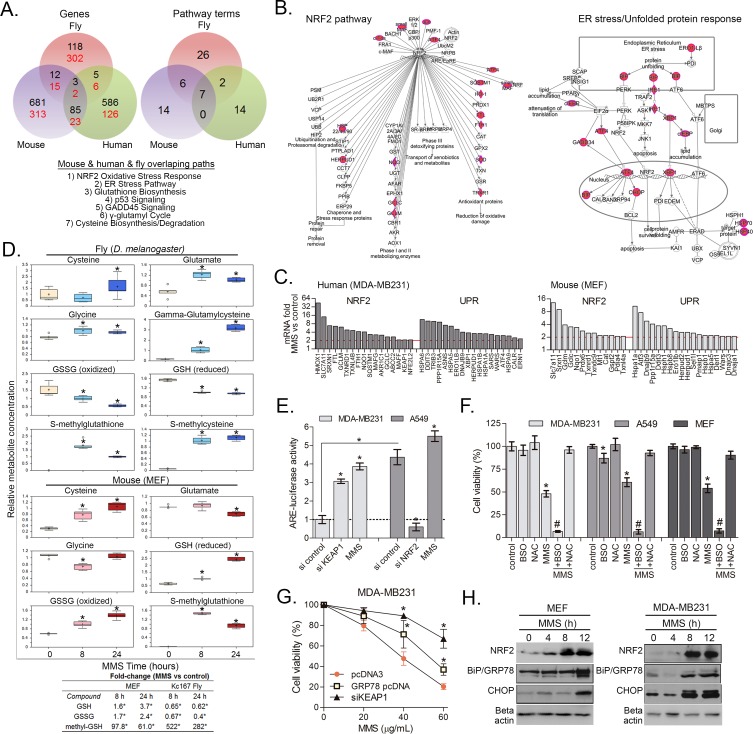
NRF2, glutathione and UPR survival responses are conserved across species. (A) Venn diagrams showing the overlap between alkylation-induced genes expressions and pathways across MDA-MB231, fly *Kc167* and MEFs. Black and red fonts denote comparisons of human and fruitfly orthologs, respectively. The pathways overlapping across the three species are also described. Detailed PEA of MMS-induced genes in MEF and MDA-MB231 are shown in S4 Table. (B) Ingenuity canonical pathway charts showing upregulated genes with the NRF2 and ER stress/UPR pathways in MMS-treated MDA-MB231 cells. Details of the edges and nodes are as described for Fig 3. (C) MMS-induced changes in NRF2 and ER stress pathway markers as determined after 8 h alkylation treatment in MDA-MB231 and MEFs. (D) Box-plot representation of MMS-induced changes in compounds of the GSH metabolism as determined by metabolomics in Kc167 and MEFs (see methods). (E) ARE luciferase assays showing the relative basal and MMS-induced NRF2 activity in MDA-MB231 and A549 cells. The effect of KEAP1 and NRF2 siRNAs is also shown as a control. (F) Cell viability assays showing the effect of NAC or BSO pre-treatments on viability of MDA-MB231, A549 and MEFs treated with ~IC50 levels of MMS for 48 h. (G)Cell viability assay showing the protective effect of KEAP1 knockdown and GRP78 chaperone overexpression upon toxicity of varying MMS levels in MDA-MB231 cells (48 h treatment). pcDNA was used as empty vector control; GRP78 overexpression (~7 fold-induction was validated by immunoblot 24 h post-transfection; data not shown); scrambled siRNA controls showed no alteration and is not shown. (H) Time course effect of MMS (40 μg/mL) on the immunocontent of NRF2, GRP78 and CHOP proteins in MDA-MB231 and MEFs as assessed by Western blot. *Different from untreated cells; ^#^different from untreated and from MMS-treated cells. In (G), asterisks denote differences from MMS alone at equivalent concentrations (ANOVA-Tukey, p<0.05, n = 3).

Having observed an enrichment of pathways involved in MMS when fusing RNAi and gene expression responses in fly cells, we wanted to determine whether we could extend this strategy to identify mammalian MMS survival pathways. Towards this objective we evaluated the integration of our findings from fly cells with MMS induced gene expression responses in normal primary mammalian MEFs and human cancer cells. We first examined the degree of overlap between MMS gene expression changes across species but found there was no consistency in the orthologs of differentially expressed genes (DEGs); there was only ~ 0.2% overlap irrespective on comparing fly (red font) human (black font) or mouse (data not shown) DEG orthologs (Fig 5A). Among ~1680 up-regulated orthologs across species only glutamate–cysteine-ligase catalytic subunit (*GCLC*), glutamine synthetase (*GLUL*) and *GADD45B* overlapped. In contrast though, pathway analysis was very informative and we were able to confirm that 7 pathway terms that can be simplified into NRF2 and GSH metabolism, p53 and GADD45 DNA damage responses and UPR/ER stress pathways are significantly conserved across species (Fig 5A; upregulated genes are detailed in S4 Table). MMS-induced genes with NRF2 and UPR/ER stress pathways in MDA-MB231 are represented in Fig 5B and MMS-induced fold-changes with NRF2 and UPR pathway markers are shown in Fig 5C. Thus, similar to our observation within *Drosophila* comparing fly RNAi screening and microarray data, comparison of gene expression responses across species is possible if conducted at a pathway/process level, more so than at an individual gene level.

Our comparative gene expression analysis again indicated the importance the NRF2-GSH and UPR pathways for alkylation survival. To confirm this result we first performed mass-spectrum metabolomics in MEFs following MMS exposure. While fly cells were unable to upregulate or maintain their GSH and GSSG levels in response to MMS, a 5 to 20-fold increase in the GSH precursor gamma-glutamyl-cysteine (gamma-GC) was observed (Fig 5D). Gamma-GC is a product of the NRF2 target enzyme *GCLC*. Interestingly, we also observed a ~300-500-fold accumulation of methyl-glutathione (methyl-GS), likely a product of phase-II detoxification of MMS by GSTs, in MMS-treated fly cells. This suggests that *de novo* GSH biosynthesis is driven by depletion of GSH pools used for MMS detoxification. This GSH response is conserved in MEFs, which seems to be better able to promote control of GSH synthesis by accumulating more GSH and GSSG (2-3-fold increases) and less methyl-GS (60 to 100-fold increase at 8 and 24 h, respectively) when compared to fly cells (Fig 5D).

MMS induced NRF2 activation in MDA-MB231 cells was first evaluated by using the ARE-luciferase reporter assay (Fig 5E). Confirming this response we demonstrated an MMS induced accumulation of NRF2 protein in MDA-MB231 and MEFs (Fig 5H–5G). Finally, to demonstrate the importance of the NRF2 pathway in MMS survival we found that we were able to promote MMS resistance by inducing NRF2; to achieve this we depleted the NRF2 negative regulator KEAP1 (Fig 5G; effect of KEAP1 siRNA on NRF2 activity is shown in Fig 5E). To complement this observation we then used the A549 lung cancer cell line, which is known to harbor an inactivating mutation in KEAP1 gene [15]. A549 cells display a constitutively increased ARE-luciferase reporter activity as compared to MDA-MB231 (Fig 5E) and an MMS IC50 level twice (75 μg/mL) than those observed in MDA-MB231 or MEF (~35 μg/mL).

To further demonstrate the importance of the NRF2/UPR pathway in alkylation survival we modulated the GSH response. GSH *de novo* biosynthesis by the GCLC/GCLM enzyme complex can be inhibited by BSO. BSO treatment caused substantial potentiation of MMS toxicity in normal MEF, MDA-MB231 as well as A549 cancer cell lines (Fig 5F). Conversely, supplementing glutathione levels by providing the rate limiting precursor cysteine in the form of NAC completely blocked MMS toxicity in all cell lines tested. As such, our results indicate that the metabolite GSH is a major requirement for MMS survival as predicted in our cross-species analysis (Fig 5F). To evaluate the ER stress/UPR response to MMS, we evaluated the ER stress sensor chaperone BIP/GRP78 and the signal transducer CHOP (Fig 5H) in MEFs and MDA-MB231. In response to MMS we observed increases in levels of both proteins. To demonstrate the importance ER stress in MMS survival we overexpressed GRP78, a key ER chaperone that inhibits ER sensor activation, which conferred protection against MMS toxicity (Fig 5G). These fly-to-mammalian data comparisons therefore confirmed the biology systems predictions that NRF2-GSH xenobiotics detoxification and UPR pathways are cross-species conserved survival responses to alkylation.

## Discussion

Forward genetic screens in model organisms have provided the basis of much of our foundational knowledge of gene function [16, 17]. The availability of RNAi platforms have provided the potential to comprehensively interrogate the role of all genes in a particular context. However, these genome scale studies result in additional complications, particularly the volume of genes being identified. Furthermore, the level of false positives (usually ~40%) and false negatives (unknown amount) [1, 18, 19] is a significant impediment to leveraging the full potential of these genomic screens. Theoretically, a complementary genomic assay could facilitate prioritization of which genes and pathways are key to the phenotype being screened. One of the most easily implemented genomic platforms currently available is gene expression analysis, be that through microarray or sequencing technologies. Conceptually, one might expect that the comparison of two or more genomic scale analyses of the same biology could help prioritize key genes for follow-up studies. However, this type of complementary analysis (gene expression plus RNAi screening data) has been explored previously to facilitate genomic knockout or RNAi screening analysis without success due to a lack of overlap between survival gene hits and those that are transcriptionally regulated in the same biological context [2, 3].

Similar to these prior reports [2, 3], we did not find a significant overlap in the genes that confer a phenotype and those that are dynamically expressed in the same context. We also noted that many of the pathways significantly enriched in either RNAi screening or gene expression analyses also largely differed. However, this did not preclude some genes being dynamically expressed in response to MMS in pathways identified from RNAi screening or conversely some genes conferring sensitivity to MMS in the pathways identified in the gene expression analysis. The fusion of the data from these two platforms therefore improved the enrichment in a majority of these pathways, including those previously validated as being necessary for MMS survival [1]. This occurs because fusion of these different data adds additional nodes to specific networks/pathways, thus highlighting which pathways are most likely to validate following further examination. Importantly, while gene level comparison across species was uninformative, fusion followed by PEA was a powerful means to compare data across evolutionary distant species such as fly to human. This observation suggests that different cells, even from different species, share similar pathways/processes to respond to alkylation damage, though modulating different individual genes (or their orthologs) within a conserved pathway to achieve this effect. Inherent to these observations is the concept that the regulation of pathway activity by the dynamic expression of a gene does not indicate that these same genes are essential to that process. Conversely, the proteins that are sensitive to RNAi knockdown and are key for pathway activity may not be the same genes in that are dynamically expressed. This is exemplified by *Hsf* and *cnc/NRF2*, two master regulators of the UPR and NRF2 responses mostly regulated at the posttranslational level [11, 20, 21], whose knockdown caused sensitivity to MMS, but gene expressions were not altered. In addition, fusion provides improved gene enrichments by better covering both gene expression changes and posttranslational-regulated RNAi hits with a given pathway and thus consists an interesting strategy for improve pathway identification that may have wider utility.

To validate the results of our fusion strategy and its utility in comparative biology analysis we focused on a relatively underexplored component of alkylation damage survival. Alkylating agents are electrophilic compounds that can react with cysteine groups such as is found in KEAP1. Once these cysteine groups are altered, KEAP1 can no longer bind NRF2 and target it for degradation. NRF2 can then accumulate and promote transcription of antioxidant and detoxification genes following its translocation into the nucleus [11, 21]. We found that NRF2 upregulates *GCLC* to replenish GSH pools that are depleted by the formation of methyl-GSH during MMS detoxification. Consequently, depletion of GSH potentiates alkylation toxicity. Substantiating our results others have reported that GSH depletion potentiates chemotherapy toxicity or that up-regulation of the NRF2 or GSH system confers chemoresistance [22–26]. Interestingly, the GSH precursor NAC blocks alkylation associated nephrotoxicity [27, 28], though our data from normal cells such as MEFs and the conservation of NRF2-GSH response would predict NAC also to cause an undesired protection of cancer cells. GSH also can facilitate chemoresistance by serving as a cofactor for MRP2-mediated drug efflux [29] or GSTs to detoxify xenobiotics such as alkylators [30]. Here, we connected NRF2 and GSH in the context of alkylating drugs response, and showed that this is a well-conserved process across evolutionary distant organisms. This raise concerns about the use of NRF2 inhibition in combination cancer therapies due to possible off-target toxicity [31].

Our results also indicated that the UPR is also key in determining alkylation survival, and enhancing the folding capability of ER via overexpression of the chaperone GRP78 conferred resistance to alkylation. Although not herein identified, alkylation of nascent proteins within the ER may be the mechanism underlying the ER stress induction by alkylators [32]. Of note, studies with the alkylation-like chemotherapeutic cisplatin suggest that it may not be widespread protein alkylation that induces UPR, but rather conjugation of key proteins such as the GRP94, HSP90 and calreticulin within the ER [32]. ER stress promotes adaptive responses or cell death, depending on the duration of the stimulus and which ER sensors are activated [32, 33]. While ATF6 and IRE1a ER sensors are first responders to unfolded proteins and generally promote production of chaperones, ER biogenesis, proteolysis and secretion, thus enhancing ER folding capacity [7, 8]. On the other hand, PERK responds to high and persistent unfolded cargo and inhibits global mRNA translation via eIF2α phosphorylation leading to apoptosis via ATF4-CHOP axis [9, 10]. The complex and differential mechanisms associated with ER sensors activation by chemotherapies requires more in depth investigation in order to understand how modulating ER stress sensors might optimize or hamper alkylation toxicity to normal or tumor contexts.

In summary, our data show that it is possible to fuse fly RNAi and gene expression data at a pathway level, but not gene level, in order to better identify cellular responses to a cytotoxic stimuli. Further we show that this fusion concept and be extended from fly to more complex transcriptomes, such as mammalian. RNAi and gene expression combination analysis (e.g. fusion) are able to differentiate dynamically from non-dynamically expressed survival responses, as well as provide a better overview and gene enrichment with pathways. Besides the classical and expected DDR pathways, we validated the activation of NRF2 and UPR as two conserved alkylation responses, which are possibly a consequence of MMS proteotoxicity and a cellular attempt of drug detoxification. These DNA repair independent mechanisms are likely relevant to both normal and cancer cell responses to alkylating agents and possibly other genotoxic chemotherapeutics. It is very likely that this fusion strategy can be applied to other contexts where both RNAi and gene expression data are available. Further, as we better develop pathway analysis tools and develop databases of genes that are either capable of dynamic expression or responsive to RNAi, we should be better able to use bioinformatics to minimize false positive results and improve potential false negative results that need to be retested.

## Supporting Information

S1 Fig Time course of MMS-induced gene expression alterations in Kc167 cells. (A) Description of MMS upregulated genes over 8, 24 and 72 h treatments in fly Kc167 cells. (B) Distribution of the 52 MMS survival hits with upregulated gene expressions over 8, 24 and 72 h treatments in fly Kc167 cells. In A and B, genes are annotated to their respective time points of upregulation. (C) Fold changes of MMS survival hits with concomitant up (52 genes) and downregulated (26 genes) expressions over 8, 24 and 72 h MMS treatment. (D) Representative pathways associated with MMS upregulated genes in 8 h compared to combined 8, 24 and 72 h gene lists shows better enrichments in combined analysis.
(TIF)

S2 Fig Venn Diagrams of pathways associated with MMS survival hits and microarray changes. Detailed Venn diagrams of Pathway terms associated with MMS induced genes (microarray), RNAi survival hits (RNAi screening) and fusion (microarray+RNAi screening). In the bottom-right graph, the antilo\g p-values of pathway enrichments in each part of Venn diagram are shown. Pathway terms overlapping between two platforms show more significant p-values than orphan terms.
(TIF)

S1 Table Differentially expressed genes in MMS-treated Kc167 cells. MMS-induced changes in gene expression of fly *Kc167* cells (8, 24 and 72 h) as determined by microarray. Essentiality of each gene for MMS survival is also annotated as previously determined by Ravi *et al*. 2009 [1].
(XLSX)

S2 Table Detailed Pathway Enrichment Analysis (PEA) of MMS-treated Kc167 cells. Pathway Enrichment Analysis (PEA) of MMS-induced genes (microarray), RNAi screening survival hits (from [1]), and fusion of microarray/RNAi screening gene lists in *Kc167 D*. *melanogaster* cell line treated with MMS.
(XLSX)

S3 Table Alkylation-induced changes in NRF2 and UPR genes in fruitfly cells. NRF2-GSH and UPR/ER stress pathway gene expression changes in *Kc167* cells treated with 40 μg/mL MMS for 8 and 24 h as determined by microarray. Essentiality of each gene for MMS survival is annotated as previously determined by Ravi *et al*. 2009 [1]
(XLSX)

S4 Table MMS-induced pathways in mouse and human cells. Pathway Enrichment Analysis (PEA; Ingenuity) of alkylation-induced gene expressions in MDA-MB231 and MEF cells treated with 40 μg/mL MMS for 8 h as determined by RNA sequencing and microarray, respectively.
(XLSX)
