# Supplementary figures and images for: Combined Gene Expression and RNAi Screening to Identify Alkylation Damage Survival Pathways from Fly to Human
Source: PLoS One. 2016 Apr 21;11(4):e0153970. doi: 10.1371/journal.pone.0153970 (PMC4839732; doi:10.1371/journal.pone.0153970)

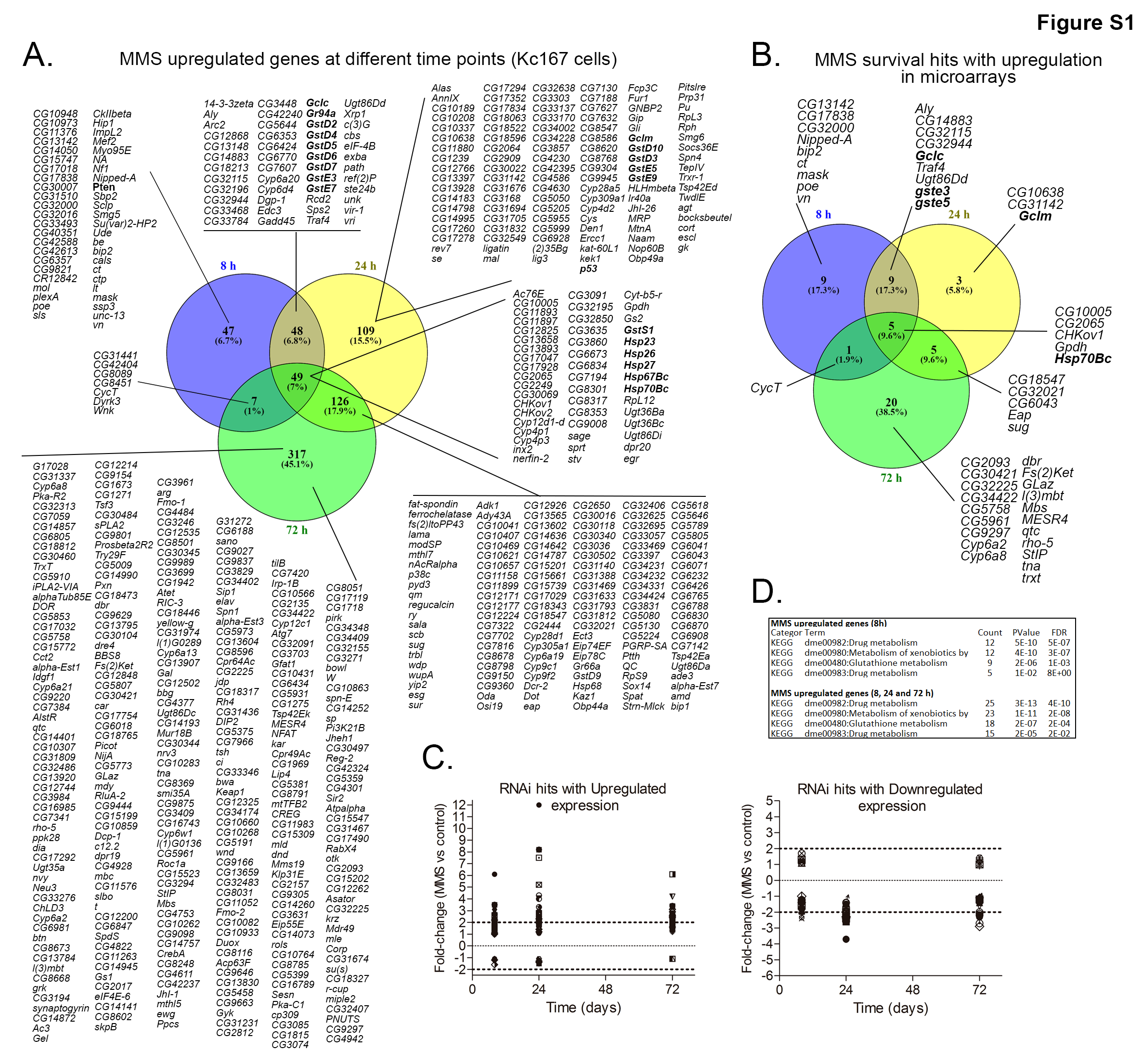

Supplement: S1 Fig — (A) Description of MMS upregulated genes over 8, 24 and 72 h treatments in fly Kc167 cells. (B) Distribution of the 52 MMS survival hits with upregulated gene expressions over 8, 24 and 72 h treatments in fly Kc167 cells. In A and B, genes are annotated to their respective time points of upregulation. (C) Fold changes of MMS survival hits with concomitant up (52 genes) and downregulated (26 genes) expressions over 8, 24 and 72 h MMS treatment. (D) Representative pathways associated with MMS upregulated genes in 8 h compared to combined 8, 24 and 72 h gene lists shows better enrichments in combined analysis. (TIF) [file pone.0153970.s001.tif]

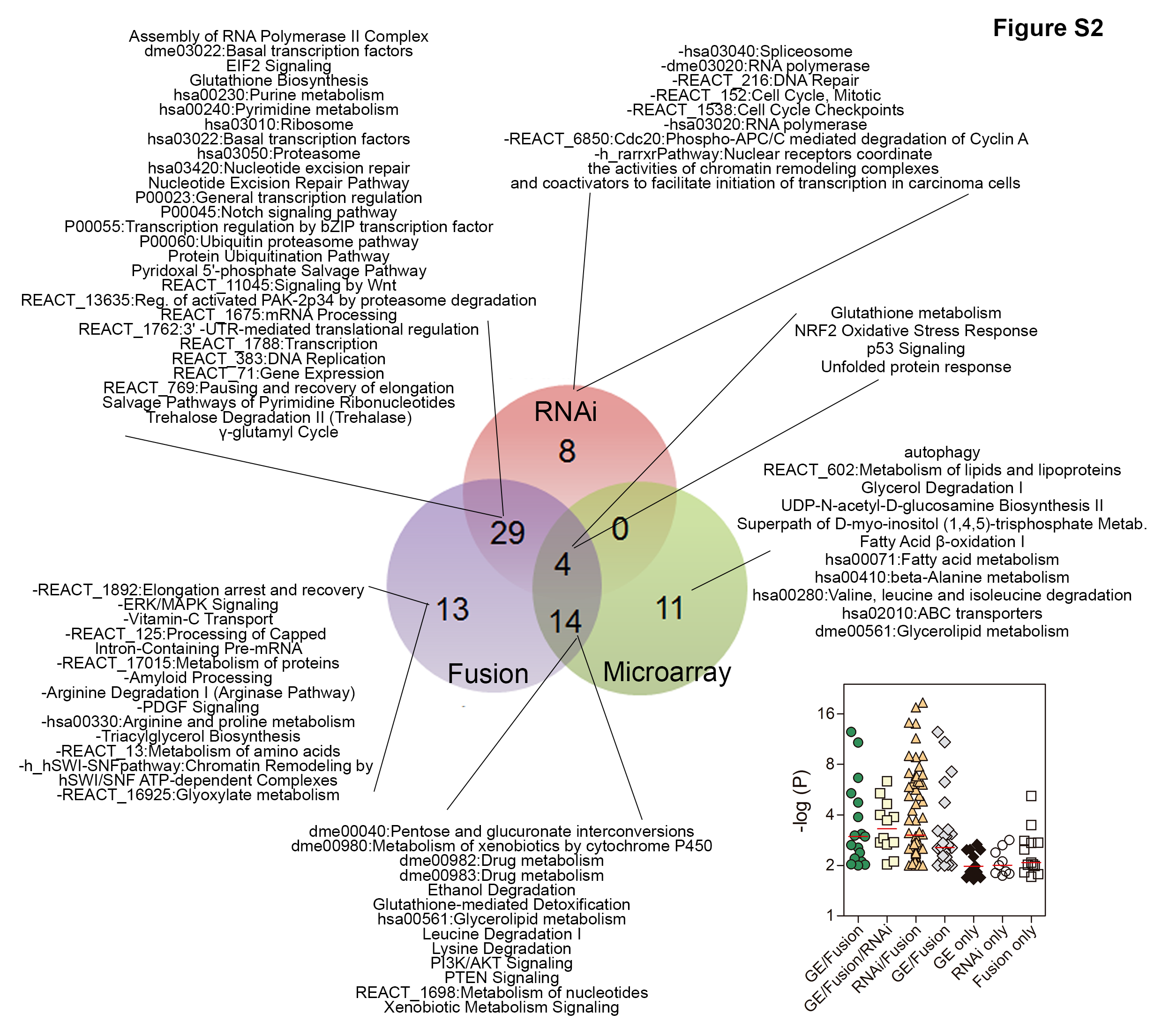

Supplement: S2 Fig — Detailed Venn diagrams of Pathway terms associated with MMS induced genes (microarray), RNAi survival hits (RNAi screening) and fusion (microarray+RNAi screening). In the bottom-right graph, the antilo\g p-values of pathway enrichments in each part of Venn diagram are shown. Pathway terms overlapping between two platforms show more significant p-values than orphan terms. (TIF) [file pone.0153970.s002.tif]
